# The Prevalence of Ocular Manifestations in the Various Types of Common Skin Disorders at Tertiary Hospital in Ahmedabad, India

**DOI:** 10.7759/cureus.25881

**Published:** 2022-06-12

**Authors:** Vijaykumar M Damor, Anupama J Gosai, Shabnam G Ipli

**Affiliations:** 1 Ophthalmology, AMC MET Medical College/ Sheth L.G. General Hospital, Ahmedabad, IND

**Keywords:** lagophthalmos, skin diseases, psoriasis, leprosy, neurofibromatosis, acne, hsv, hzo, prevalence, ocular manifestations

## Abstract

Aim

The article aims to study the prevalence and ocular manifestations of the various types of common skin disorders at a tertiary hospital in Ahmedabad, India.

Materials and methods

Five hundred patients were studied in the ophthalmology department from September 2017 to September 2019.

Results

Out of the 500 patients that were included in our study, 312 were males, and 188 were females. In our study, lid involvement was seen most commonly in patients with herpes zoster ophthalmicus (HZO). Plexiform neurofibroma was seen in eight (14.5%) patients with neurofibromatosis (NF). Cicatricial ectropion was found in half of the patients with ichthyosis. Lagophthalmos was present in 10 (20.4%) patients with leprosy. Conjunctivitis was seen commonly in HZO patients. Papillary conjunctivitis was seen in half of the atopic dermatitis cases. Steven Johnson's syndrome (SJS) was associated with pseudomembranous conjunctivitis. Conjunctival xerosis was seen only in patients with Sjogren's syndrome. The cornea was most commonly involved in patients of HZO. Decreased corneal sensation was seen in leprosy and HZO. Lisch nodules were seen in NF cases. Anterior uveitis was found in seven (6.3%) patients with HZO. Glaucoma was seen most commonly in patients with Sturge Weber syndrome (SWS), followed by leprosy. Facial nerve palsy was seen in patients with leprosy. Episcleritis was seen in patients with leprosy and SWS.

Conclusion

We conclude that ocular involvement in skin disease is a common feature and could be a major component of the development of various systemic skin disorders.

## Introduction

Dermatology disorders can have numerous ocular manifestations. Common dermatological disorders can manifest with mild to severe ocular manifestations that can result in ocular damage, and sometimes it can even cause vision loss. Dermatology patients are prescribed long-term medications like steroids, hydroxychloroquine, retinoic acid, immunosuppressive agent, which can cause indirect side effects on the eyes and is one of the leading causes of ocular manifestations in dermatology patients. So thorough examination, ophthalmological referral, and long-term follow-up of dermatology patients are paramount important. The early detection of the clinical relationship between ocular manifestations and dermatological disease is important for the proper management of the patient because many dermatology diseases can manifest initially with ocular findings. In our study, we included the most common and significant ocular manifestations in major dermatological diseases, which should be studied and analyzed for further understanding and management of the patients. 

Herpes zoster ophthalmicus (HZO) is characterized by an eruption of multiple vesicles strictly on one side of the face along with the distribution of the ophthalmic division of the trigeminal nerve, preceded by severe neuralgia and constitutional symptoms. HZO is caused by the varicella-zoster virus, which reactivated from its dormant status in the dorsal ganglion cells of the central nervous system [1]. An endogenous reactivation of latent virus occurs in elderly persons. Mild cases of HZO may develop conjunctivitis, superficial punctate keratitis, and subepithelial infiltrates, while severe cases include sclerokeratitis, interstitial keratitis, and neurotrophic keratopathy. 

Herpes simplex virus (HSV) type 1 causes ocular manifestations like herpes keratitis, stromal keratitis, iridocyclitis, and acute retinal necrosis in older patients leading to visual impairment. Conventionally, the diagnosis of HSV keratitis is based on a history of recurrent keratitis, as well as typical clinical manifestations in the infected eye [2]. Primary herpetic infection is found in non-immune persons. The primary infection may take a mild or a fatal course if encephalitis develops. The disease may cause mild fever, malaise, and non-suppurative preauricular lymphadenopathy. The infection remains subclinical in affected persons. Reactivation of the virus occurs following poor general body resistance in conditions like debilitating diseases, stress, use of corticosteroids and immunosuppressive agents. In Toma et al. study, HSV-1 DNA was found in 93% of human trigeminal ganglia [3].

Ocular rosacea is a chronic inflammatory condition that affects the skin and oil glands. Meibomian glands in the eyelid, which produce the oil component of the tear film, are affected in rosacea. Ocular manifestations include chronic red eyes, styes, chalazia, and severe photophobia. Patients can develop infiltrative marginal keratitis and scarring of the cornea. It involves more than 50% of patients with dermatology diseases [4].

Neurofibromatosis (NF) is a type of phacomatosis with a genetic abnormality that affects neural tissue growth and affects the nervous system, skin, eyes, and other organs. Genetic abnormalities are either passed on by parents or occur spontaneously at conception. NF is divided into two primary subgroups: type 1 (NF1) peripheral neurofibromatosis and type 2 (NF2) central neurofibromatosis. Eyelid neurofibroma is a type of phacomatosis where progressively fast-growing lid tumor tends to develop early in younger age groups, obscures the visual axis, and eventually affects the vision of the patient.

Leprosy or Hansen's disease is a chronic infectious disease caused by an intracellular rod-shaped acid-fast bacilli Mycobacterium leprae which affects the skin, nasal mucosa, peripheral nerves, and the anterior segment of the eye [5]. Ocular manifestations include cataract, lagophthalmos, reduced corneal sensations, exposure keratopathy, and uveitis. A corneal ulcer may occur due to an acute microbial infection or as a secondary infection in exposure keratitis.

Psoriasis is an uncommon disease that often involves the eye. It affects males and females equally [6]. Skin can present with persistent scaly plaques, flaking, itch, and pain. The ocular manifestations include conjunctivitis, chronic blepharitis, keratitis, trichiasis, symblepharon, anterior uveitis, and retinal vasculitis. Uveitis in psoriasis patients is chronic, a bilateral condition found in older age groups, while human leukocyte antigen B27 (HLA B27) anterior uveitis is unilateral with potent posterior ocular involvement. Prolonged courses of systemic corticosteroids for psoriasis management may cause posterior subcapsular cataracts.

This article aims to study the prevalence of ocular manifestations in different types of dermatology diseases at a general hospital in Ahmedabad, India.

## Materials and methods

Our study is an observational retrospective study carried out on patients with dermatological diseases who were presented to the ophthalmology department in a tertiary hospital from September 2017 to September 2019. Patients were treated with comprehensive combined care with the dermatology department. We obtained approval from the Institutional Review Board of AMC MET Medical College, affiliated with Gujarat University, and written consent from the 500 patients with dermatology diseases with ocular manifestations we studied. Patients, who were unwilling to participate in the study and patients suffering from any mental, neurological, or debilitating illness, which hampers examination, were excluded from the study. Age, sex, and type of skin disease were noted. Patients having skin disease were examined clinically to rule out any associated ocular condition. After consulting with the dermatology department, all dermatological findings and treatments were noted. The past history of the patient was recorded, including any medications for skin disease and the duration of a particular disease. Best-corrected visual acuity of the patient was taken by Snellen's chart. Intraocular pressure of both eyes was measured in all indicated patients. In any cases of corneal epithelial defects or corneal erosions, corneal staining was done by using 2% fluorescein strips. In all cases of dry eyes, we have done Schirmer's test for five minutes using Whatman® number 41 filter paper. Those patients who are suspected of having glaucoma were sent for investigations like perimetry and optical coherence tomography. If needed, radiological investigations like X-ray orbit, magnetic resonance imaging, and computed tomography scan were done. The number of patients with a particular disease having specific ophthalmic manifestations was calculated and analyzed.

## Results

In this retrospective study, 500 patients having dermatological diseases were included, of which 312 (62.4%) were males and 188 (37.6%) were females. Ophthalmological findings were correlated with particular dermatological diseases. One hundred and eleven (22.2%) patients had HZO, 60 (12%) patients had HSV, 57 (11.4%) patients had acne, 55 (11%) patients had NF, 49 (9.8%) patients had leprosy and 35 (7%) patients had psoriasis (Table 1).

**Table 1:** Sex distribution of dermatology diseases

	Dermatology Diseases																	
	Acne	Atopic dermatitis	Steven Johnson's syndrome (SJS)	Pemphigoid	Sjogren’s syndrome	Herpes zoster ophthalmic (HZO)	Syphilis	Human immunodeficiency virus (HIV)	Neurofibromatosis (NF)	Tuberous sclerosis	Sturge Weber syndrome (SWS)	Ichthyosis	Leprosy	Systemic lupus erythematosus (SLE)	Hyper IgE	Psoriasis	Herpes simplex virus (HSV)	Total
Male	32	6	4	7	2	60	21	28	37	1	2	14	31	2	0	25	40	312
Female	25	5	1	5	1	51	12	6	18	2	1	10	18	2	1	10	20	188
Total	57	11	5	12	3	111	33	34	55	3	3	24	49	4	1	35	60	500

In our study, most patients with ocular manifestations were middle-aged: 150 (30%) from the 21-30 age group and 85 (17%) patients from the 31-40 age group. HZO was most commonly seen in elder age groups: 52 (46.8%) patients from the 51-60 age group and 55 (49.5%) from the 61-70 age group. HSV was seen in 29 (48.3%) patients in the 61-70 age group. Acne was seen in 42 (73.6%) patients in the younger age group of 21-30 years. NF, leprosy, and psoriasis were mostly seen in younger age groups. Atopic dermatitis was seen in 10 (90%) patients belonging to the 1-10 age group. Ichthyosis was seen in 22 (91.6%) patients in the 1-10 age group (Table 2).

**Table 2:** Age group distribution of dermatology diseases

Age distribution in years	Dermatology diseases																	
	Acne	Atopic dermatitis	Steven Johnson's syndrome (SJS)	Pemphigoid	Sjogren’s syndrome	Herpes zoster ophthalmic (HZO)	Syphilis	Human immunodeficiency virus (HIV)	Neurofibromatosis (NF)	Tuberous sclerosis	Sturge Weber syndrome (SWS)	Ichthyosis	Leprosy	Systemic lupus erythematosus (SLE)	Hyper IgE	Psoriasis	Herpes simplex virus (HSV)	Total
1-10	0	10	4	3	0	0	0	0	0	0	0	22	0	0	0	2	0	41
11-20	6	0	1	6	0	0	3	0	10	1	2	2	7	1	1	3	2	45
21-30	42	1	0	3	0	0	13	18	28	1	0	0	12	3	0	19	10	150
31-40	9	0	0	0	0	0	13	14	17	1	0	0	18	0	0	9	4	85
41-50	0	0	0	0	2	4	4	2	0	0	1	0	8	0	0	0	0	21
51-60	0	0	0	0	1	52	0	0	0	0	0	0	4	0	0	2	15	74
61-70	0	0	0	0	0	55	0	0	0	0	0	0	0	0	0	0	29	84
Total	57	11	5	12	3	111	33	34	55	3	3	24	49	4	1	35	60	500

In our study, the most common lid manifestations were vesicles seen in 91 (81.9%) patients, and scarring was seen in 10 (9.0%) HZO patients. In acne patients, blepharitis, meibomian gland dysfunction (MGD), and stye were seen in 22 (38.5%), four (7%), and five (8.7%) patients, respectively. Plexiform NF was seen in eight (14.5%) patients. Ninety-two (18.4%) patients had conjunctivitis. Three (60%) patients with Steven Johnson's syndrome (SJS) had pseudo membrane. Two (66.6%) patients with Sjogren's syndrome had conjunctival xerosis (Table 3).

**Table 3:** Eyelid and conjunctival findings in dermatology diseases MGD - meibomian gland dysfunction; NF - neurofibromatosis

Eyelid finding	Dermatology diseases																	
	Acne	Atopic dermatitis	Steven johnson'S syndrome (SJS)	Pemphigoid	Sjogren's syndrome	Herpes zoster ophthalmicus (HZO)	Syphilis	Human immunodeficiency virus (HIV)	Neurofibromatosis (NF)	Tuberous sclerosis	Sturge Weber syndrome (SWS)	Ichthyosis	Leprosy	Systemic lupus erythematosus (SLE)	Hyper IgE	Psoriasis	Herpes simplex virus (HSV)	Total
Blepharitis	22	1	0	0	0	2	0	0	0	0	0	0	0	0	0	0	0	25
Chalazion	2	0	0	0	0	0	0	0	0	0	0	0	0	0	0	0	0	2
Crusting	0	0	4	0	0	0	0	0	0	0	0	0	0	0	0	0	0	4
Ectropion	0	0	0	0	0	0	0	0	0	0	0	12	0	0	0	0	0	12
Lagophthalmos	0	0	0	0	0	0	0	0	0	0	0	0	10	0	0	2	0	12
MGD	4	0	0	0	0	0	0	0	0	0	0	0	0	0	0	0	0	4
Plexiform NF	0	0	0	0	0	0	0	0	8	0	0	0	0	0	0	0	0	8
Scarring	0	0	0	0	0	10	0	0	0	0	0	0	0	0	0	0	0	10
Stye	5	0	0	5	0	2	0	0	0	0	0	0	0	0	0	0	0	12
Vesicles	0	0	0	0	0	91	0	0	0	0	0	0	0	0	0	0	0	91
Normal	24	10	1	7	3	6	33	34	47	3	3	12	39	4	1	33	60	320
Total	57	11	5	12	3	111	33	34	55	3	3	24	49	4	1	35	60	500
Conjunctival findings																		
Allergic keratoconjuctivitis	0	0	0	0	0	0	0	0	0	0	0	0	0	0	1	0	0	1
Conjunctival xerosis	0	0	0	0	2	0	0	0	0	0	0	0	0	0	0	0	0	2
Conjunctivitis	0	0	1	6	0	34	11	12	0	0	0	8	0	2	0	12	06	92
Papillary conjunctivitis	0	5	0	0	0	0	0	0	0	0	0	0	0	0	0	0	0	5
Pseudo membrane	0	0	3	0	0	0	0	0	0	0	0	0	0	0	0	0	0	3
Normal	57	6	1	6	1	77	22	22	55	3	3	16	49	2	0	23	54	397
Total	57	11	5	12	3	111	33	34	55	3	3	24	49	4	1	35	60	500

In our study, keratitis was seen in 29 (26.1%) HZO patients, while decreased corneal sensation was seen in 14 (28.5%) leprosy patients. Anterior uveitis was seen in seven (6.3%) patients with HZO, while lisch nodules were seen in 41 (74.5%) patients with NF (Table 4).

**Table 4:** Cornea and uvea findings in dermatology diseases KPs - keratic precipitates

Corneal findings	Dermatology diseases																	
	Acne	Atopic dermatitis	Steven Johnson syndrome (SJS)	Pemphigoid	Sjogren’s syndrome	Herpes zoster ophthalmicus (HZO)	Syphilis	Human immunodeficiency virus (HIV)	Neurofibromatosis (NF)	Tuberous sclerosis	Sturge Weber syndrome (SWS)	Ichthyosis	Leprosy	Systemic lupus erythematosus (SLE)	Hyper IgE	Psoriasis	Herpes simplex virus	Total
Decreased corneal sensation	0	0	0	0	0	0	0	0	0	0	0	0	8	0	0	0	0	8
Epithelial keratitis, endotheliitis	0	0	0	0	0	0	0	0	0	0	0	0	6	0	0	0	0	6
Endotheliitis	0	0	0	0	0	5	0	0	0	0	0	0	0	0	0	0	0	5
Epithelial keratitis	0	0	0	0	0	18	0	0	0	0	0	0	0	0	0	0	10	28
Epithelial keratitis, Hutchison's sign	0	0	0	0	0	6	0	0	0	0	0	0	0	0	0	0	0	6
KPs	0	0	0	0	0	8	0	0	0	0	0	0	0	0	0	0	0	8
Megalocornea	0	0	0	0	0	0	0	0	0	0	3	0	0	0	0	0	0	3
Opacification	0	0	1	0	0	0	0	0	0	0	0	0	0	0	0	0	0	1
Stromal keratitis	0	0	0	0	0	3	0	0	0	0	0	0	0	0	1	0	0	4
Normal	57	11	4	12	3	71	33	34	35	3	0	24	35	4	1	35	50	431
Total	57	11	5	12	3	111	33	34	55	3	3	24	49	4	1	35	60	500
Uveal findings																		
Anterior uveitis	0	0	0	0	0	7	0	0	0	0	0	0	0	0	0	7	0	14
Hetero chromia	0	0	0	0	0	0	0	0	0	0	2	0	0	0	0	0	0	2
Iris pearls	0	0	1	0	0	0	0	0	0	0	0	0	0	0	0	0	0	1
Lisch nodules	0	0	0	0	0	0	0	0	41	0	0	0	0	0	0	0	0	41
Normal	57	11	4	12	3	104	33	34	14	3	1	24	49	4	1	28	60	442
Total	57	11	5	12	3	111	33	34	55	3	3	24	49	4	1	35	60	500

In our study, lens manifestations were seen as cataract in 136 (27.2%) patients. Out of these, 86 (77.4%), 32 (53.3%), and 15 (27.2%) were in patients with HZO, HSV, and leprosy, respectively. Posterior subcapsular cataract was present in eight (16.3%) patients with leprosy. Astrocytoma, retinal manifestation, was seen in one patient with tuberous sclerosis (TS). Fifteen (3%) patients had glaucoma, out of which 11 (22.4%) were seen in patients with leprosy. Ocular manifestation, like seventh cranial nerve palsy, was seen in 14 (28.5%) patients with leprosy, and episcleritis was seen in 10 (20.4%) patients with leprosy (Table 5). 

**Table 5:** Lens, retina, optic nerve head and other findings in dermatology diseases

Lens findings	Dermatology diseases																	
	Acne	Atopic dermatitis	Steven Johnson syndrome (SJS)	Pemphigoid	Sjogren’s syndrome	Herpes zoster ophthalmicus (HZO)	Syphilis	HIV	Neurofibromatosis (NF)	Tuberous sclerosis	Sturge Weber syndrome (SWS)	Ichthyosis	Leprosy	Systemic lupus erythematosus (SLE)	Hyper IgE	Psoriasis	Herpes simplex virus	Total
Anterior subcapsular cataract	0	1	0	0	0	0	0	0	0	0	0	0	0	0	0	0	0	1
Cortical cataract	0	0	0	0	2	37	0	0	0	0	0	0	1	0	0	0	16	56
Nuclear cataract	0	0	0	0	0	49	0	0	0	0	0	0	15	0	0	0	16	80
Nuclear cataract, cortical cataract	0	0	0	0	0	0	0	0	0	0	0	0	3	0	0	0	0	3
Posterior subcapsular cataract	0	0	0	0	0	0	0	0	0	0	0	0	8	0	0	0	0	8
Pseudophakia	0	0	0	0	0	25	0	0	0	0	0	0	0	0	0	0	8	33
Normal	57	10	05	12	1	0	33	34	55	3	3	24	22	4	1	35	20	319
Total	57	11	05	12	3	111	33	34	55	3	3	24	49	4	1	35	60	500
Retinal findings																		
Astrocytoma	0	0	0	0	0	0	0	0	0	1	0	0	0	0	0	0	0	1
Absent	57	11	5	12	3	111	33	34	55	2	3	24	49	4	1	35	60	499
Total	57	11	5	12	3	111	33	34	55	3	3	24	49	4	1	35	60	500
Optic nerve head findings																		
Glaucomatous	0	1	0	0	0	0	0	0	0	0	3	0	11	0	0	0	0	15
Normal	57	10	5	12	3	111	33	34	55	3	0	24	38	4	1	35	60	485
Total	57	11	05	12	3	111	33	34	55	3	3	24	49	4	1	35	60	500
Other ocular findings																		
Seventh cranial nerve palsy	0	0	0	0	0	0	0	0	0	0	0	0	14	0	0	0	0	14
Episcleritis	0	0	0	0	0	0	0	0	0	0	3	0	10	0	0	0	0	13
Normal	57	11	5	12	3	111	33	34	55	3	0	24	25	4	1	35	60	473
Total	57	11	5	12	3	111	33	34	55	3	3	24	49	4	1	35	60	500

## Discussion

In this retrospective study of patients having dermatological diseases total of 500 patients were included, of which 312 (62.4%) were males, and 188 (37.6%) were females. Ophthalmological findings were correlated with particular dermatological diseases. One hundred and eleven (22.2%) patients had HZO, 60 (12%) patients had HSV, 57 (11.4%) patients had acne, 55 (11%) patients had NF, 49 (9.8%) patients had leprosy, and 35 (7%) patients had psoriasis.

In our study, lens manifestations were seen as cataract in 136 (27.2%) patients. Out of these, 86 (77.4%), 32 (53.3%), and 15 (27.2%) had HZO, HSV, and leprosy, respectively, which was due to patients from older age group.

Of 111 patients with HZO, 60 (54.05%) patients were males, and 51 (45.9%) were females, with a male to female ratio of 1.17:1. A similar finding was seen in the study by Dubey et al. in South India, where the male to female ratio was l.84:1 [7]. Sehgal et al. also noted that males are affected more than females [7-9]. In our study, the commonest age group of presentation among the patients with HZO was 51-60 years. HZO was most commonly seen in elder age groups: 52 (46.8%) patients from the 51-60 age group and 55 (49.5%) from the 61-70 age group. Sehgal et al. found a high incidence in the fourth and fifth decades [8]. In our study, the most common lid manifestations were vesicles seen in 91 (81.9%) patients and scarring seen in 10 (9.0%) patients with HZO, similarly to the study conducted by Nigran et al. [9]. In our study, anterior uveitis was seen in seven (6.3%) patients with HZO. Herpetic anterior uveitis was the most common cause of viral anterior uveitis accounting for 5-10% of all uveitis cases in the western world and 0.9-8.3% of all infectious uveitis in India [10].

A total of the 60 patients had the herpes simplex virus; 40 (66.6%) patients were males, and 20 (33.3%) patients were females giving a male to female ratio of 2:1. A study conducted by Kaul et al. in North India estimated the incidence of HSV1 as 33.3% [11], while in our study, the incidence of HSV1 was 12.0%. In our study, epithelial keratitis was seen in 10 (16.6%) patients with HSV, which corresponds with the Fukuda et al. study reporting the highest number of copies of HSV-DNA in herpetic epithelial keratitis, followed by active stromal keratitis and persistent epithelial defect. Their detection rate was higher at 88.1% for epithelial keratitis and 59.1% for stromal keratitis [12].

A total of 57 (11.4%) patients had acne; 32 (56.1%) patients were males, and 25 (43.8%) patients were females giving a male to female ratio of 1.28:1. Ocular rosacea affects both males and females equally [13]. Ocular rosacea was seen in more than half of the patients with dermatology disease, indicating a high prevalence rate of ocular manifestation [14]. Rosacea most commonly affects middle-aged adults [15]. The mean age in our study was found to be 25 years. Lid inflammation and oil gland dysfunction are common findings in patients with ocular rosacea leading to dry eye [16].

A total of the 55 patients had neurofibromatosis; 37 (67.27%) were males, and 18 (37.2%) were females. This corresponds with the study conducted by Odebode et al., in which there was a definite male predominance, affecting 60 males and 38 females with a total of 98 patients [17]. The commonest age group with 45 (81.8%) patients presented to us was between 20-40 years. In our study, eight (14.5%) of the 55 patients with neurofibromatosis had plexiform neurofibroma, while lisch nodules were the commonest manifestation present in 41 (74.5%) patients [18].

Of the 49 patients with leprosy, 31 (63.2%) were males, and 18 (36.7%) were females. The male to female ratio is 1.72:1 [19]. There is no significant difference in the age distribution of patients with Hansen's disease as compared to that of patients with ocular involvement. The majority of the patient with ocular involvement was seen in the age group of 21-40 years [20]. In the study conducted by Moschella,** **out of 250 cases, 148 (59.2%) cases showed ocular lesions [21]. This variation can be racial too, Asiatics being more susceptible. In our study, 49 (9.8%) patients with leprosy had ocular manifestations. Lagophthalmos, decreased corneal sensation, epithelial endotheliitis, cataract, glaucomatous optic disc changes, seventh cranial nerve palsy, and episcleritis was seen in 10 (20.4%), eight (16.3%), six (12.2%), 27 (55.1%), 11 (22.4%), 14 (28.5%), 10 (20.4%) patients, respectively. In a study by Grzybowski et al., the major sight-threatening lesions included cataract (65%), lagophthalmos (12%), keratitis (13%), and glaucoma (9%), corresponding with our study findings [22].

A total of the 35 patients with psoriasis, 25 (71.4%) patients were males, and 10 (28.5%) were females giving a male to female ratio of 2.5:1. There is a slight male predominance, probably due to better access of male patients to healthcare services in our country. This finding is similar to the study reported earlier [23]. Among the 35 patients with psoriasis, in our study, we found conjunctivitis to be the most common manifestation seen in 12 (34.2%) patients.Seven (20%) patients had chronic anterior uveitis. Catsaru-Catsari et al. found that blepharoconjunctivitis was the most common ocular manifestation of psoriasis [24].

Atopic dermatitis was seen in 10 (90%) patients in the 1-10 age group.** **Ichthyosis was seen in 22 (91.6%) in the 1-10 age group. Three (60%) patients with SJS had pseudo membrane. Two (66.6%) patients of Sjogren's syndrome had conjunctival xerosis. Cicatricial ectropion was found in half of the patients with ichthyosis [25].

## Conclusions

In our study, the most common skin diseases associated with ophthalmic manifestations were herpes zoster ophthalmicus, herpes simplex virus, ocular rosacea, neurofibromatosis, leprosy, and psoriasis. Viral skin diseases are most commonly associated with ocular manifestations. Viral skin diseases are common and potentially devastating diseases that demonstrate significant ophthalmic morbidity if not adequately diagnosed and treated.

The majority of the ocular manifestations are seen in the anterior segment of the eye. Among phakomatosis, neurofibromatosis is associated with lisch nodules and plexiform neurofibromas. Meticulous slit-lamp examination for the presence of lisch nodule is a simple, non-invasive, inexpensive method of diagnosing neurofibromatosis accurately. As the majority of dermatological diseases are associated with ocular features, complete ocular evaluation is necessary for every patient with dermatological disease.
